# A model to predict anti-tuberculosis activity: value proposition for marine microorganisms

**DOI:** 10.1038/ja.2016.87

**Published:** 2016-07-13

**Authors:** Miaomiao Liu, Tanja Grkovic, Lixin Zhang, Xueting Liu, Ronald J Quinn

**Affiliations:** 1 Eskitis Institute for Drug Discovery, Griffith University, Brisbane, QLD, Australia; 2 Chinese Academy of Sciences, Key Laboratory of Pathogenic Microbiology and Immunology, Institute of Microbiology, Chinese Academy of Sciences, Beijing, China; 3 University of Chinese Academy of Sciences, Beijing, China

## Abstract

The development of new antibiotics effective against all strains of tuberculosis (TB) is needed. To evaluate the potential of marine microbe-derived natural products as anti-TB leads, we analyzed and compared the physico-chemical properties of 39 current TB drugs and candidates against 60 confirmed mycobacteria-active natural products. We showed that anti-TB natural products sourced from marine microbes have a large overlap with TB drug-like space. A model to predict potential anti-TB drugs is proposed.

## Introduction

Tuberculosis (TB) is responsible for the death of millions of people every year and continues to claim more lives than any other single infectious agent.^1, 2^ The high incidence of HIV—TB co-infection, together with the emergence of multi-drug resistant and extensively-drug resistant forms, has resulted in TB being one of the most significant threats to global health. Major problems limiting the development and effectiveness of new drugs to combat TB are the profound innate resistance of *Mycobacterium tuberculosis* to host defense mechanisms, as well as its intrinsic tolerance to chemotherapeutic reagents.^3^ Mycobacteria are unusually successful in surviving the presence of toxic compounds because they produce effective permeability barriers, comprising of the outer membrane and the mycolate-containing cell wall on the cell surface.^4^ As a result, among hundreds of antibiotics commercially available, anti-TB treatment is still limited to drugs that were developed >50 years ago, such as ethionamide (1960), ethambutol (1961), capreomycin (1963) and rifampicin (1963). Insufficient drug options, long treatment regimens and patient non-compliance have led to worldwide emergence of strains resistant to almost all available drugs.^5^

**Figure 1:**
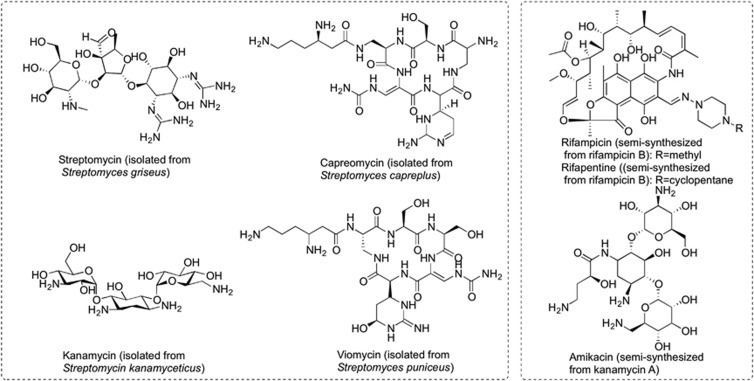
Seven TB drugs sourced or based on the NP scaffold isolated from a microbe. TB, tuberculosis.

In the search for novel drug candidates, including anti-TB drugs, natural products are evolutionary selected and pre-validated by nature, displaying unique chemical diversity and a corresponding diversity of biological activities.^6, 7^ Among the 11 currently used nature-derived TB drugs, 7 of them were either isolated from microbes or semi-synthesized from microbial natural products (Figure 1).

Traditionally terrestrial microorganisms were explored as a source of biologically active natural products, however, natural products sourced from the marine environment are becoming increasingly important as a source of structurally novel and biologically active compounds.^8, 9, 10, 11, 12, 13^ In order to examine the potential of marine microbe-sourced natural products as effective TB agents, we conducted a comprehensive literature search covering the period up to December 2014. In total, 60 marine microbial compounds have been reported to show anti-*M. tuberculosis* activity in *in vitro* models. These compounds coupled with 39 current TB drugs and candidates were used to establish a predictive model for anti-TB activity. In this period, 336 marine natural products that have not been screened for anti-mycobacterial activity were isolated from various marine microbes.

Three data sets (Table 1), TB drugs and candidates (39 compounds), mycobacteria-active natural products from marine microbes (60 compounds) and *M. tuberculosis*-untested natural products from marine microbes (336 compounds), were evaluated in this study.

Oral bioavailability will very likely increase compliance, lack of which is a major problem especially with the long duration of therapy currently required for available therapies. The lipophilicity is clearly not required to cross the mycolic acid-rich cell wall, given the good activity of several RNA-active aminoglycosides.

## Comparison of the chemGPS-NP chemical space between marine microbial natural products and TB drugs

Experimentally validated data in data sets 1 and 2 (Table 1) were used to examine the physico-chemical profiles of 39 TB drugs and candidates, and 60 mycobacteria-active natural products. Chemical global positioning system-natural product (ChemGPS-NP) is a principal component analysis-based global chemical positioning system tuned for exploration of biologically relevant chemical space, that is, those areas of chemical space most likely to enclose biologically active compounds.^14^ In ChemGPS-NP aspects of size, shape, lipophilicity, polarity, polarizability, flexibility, rigidity and hydrogen bond capacity are compared.^15^ The ChemGPS-NP space map coordinates are *t*-scores from principal component analysis using a carefully selected subset of 35 descriptors,^16^ which are then analyzed by eight respective principal components that can be mapped onto a consistent eight-dimensional map. The four most significant principle components (PCs) explain 77% of the variance and can be interpreted as follows: PC1 represents size, shape and polarizability, PC2 corresponds to aromatic- and conjugation-related properties, PC3 describes lipophilicity, polarity and H-bond capacity, and PC4 expresses flexibility and rigidity.^17^ Any compound with a known chemical structure can be positioned onto this map using interpolation in terms of principal component analysis score prediction. From the results, the properties of the compounds can be compared and easily interpreted together with trends and clusters.

**Figure 2:**
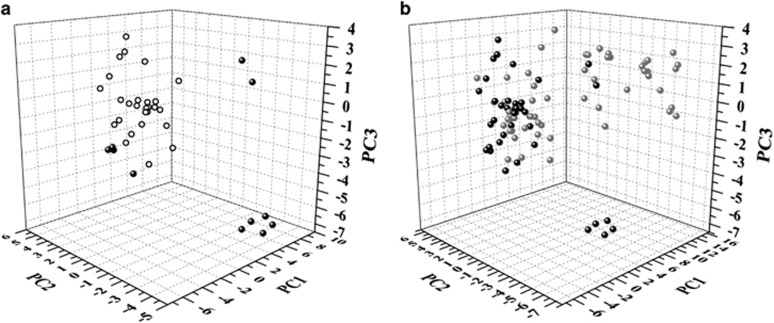
Score plot of mycobacteria-active natural products sourced from marine microbes and TB drugs. (**a**) PC1 (molecular size) versus PC2 (molecular aromaticity) versus PC3 (molecular lipophilicity) for sets of 28 synthetic TB drugs and candidates (black empty circle) and 11 nature-derived TB drugs and candidates (black filled circle) and (**b**) PC1 (molecular size) versus PC2 (molecular aromaticity) versus PC3 (molecular lipophilicity) for sets of 39 TB drugs and candidates (black) and 60 mycobacteria-active natural products from marine microbes (gray). TB, tuberculosis.

The 39 TB drugs and candidates can be divided into two groups according to their biological origin: 11 nature-derived compounds and 28 synthetic compounds. Interestingly, these two groups showed significant differences in the ChemGPS analysis (Figure 2a). Nature-derived TB drugs occupy a very broad range of physico-chemical space while most of the synthetic TB drugs distributed in the positive PC2 direction (high aromaticity). Furthermore, the nature-derived drugs and candidates can be divided into three sub-groups in the PC1/PC2/PC3 plot. The first subgroup consists of the injectable drugs, kanamycin, amikacin, streptomycin, capreomycin and viomycin, which showed high molecular weight, the least aromaticity and the lowest lipophilicity. The second subgroup is representative of low molecular weight but high aromaticity, including cycloserine, p-aminosalicyclic acid, pyrazinamide and isoniazid. The last two rifamycin compounds, rifapentine and rifampicin, belong to the third subgroup, possessing the highest molecular weight and high lipophilicity.

In this model, the mycobacteria-active natural products from marine microbes are largely overlapping with known drugs, at least in the first three dimensions (Figure 2b), strongly arguing that natural products from marine microbes have the potential to serve as an important source for TB drugs. However, 13 mycobacteria-active natural products in the upper right corner, which have higher molecular size, relative low aromaticity and are rather non-polar, do not coincide with any of the known drugs or candidates. These compounds may have a new mode of action for TB inhibition.

The area containing the TB drugs, drug candidates and mycobacteria-active natural products was subdivided into 27 regions in the ChemGPS-NP according to the values of the first three most significant PCs (explaining 71% of the variance). Each of the regions were analyzed in terms of occupancy with regard to both chemical properties and biological activities of the compounds. The scores specification of each region is listed in Table 2.

The basic interpretation of the 27 regions of ChemGPS-NP is as follows: the size of compounds increases in the positive direction of PC1 (that is, small molecules: regions 1, 4, 7, 10, 13, 16, 19, 22 and 25; medium size molecules: regions 2, 5, 8, 11, 14, 17, 20, 23 and 26; large molecules: regions 3, 6, 9, 12, 15, 18, 21, 24 and 27); compounds are increasingly aromatic in the positive direction of PC2 (that is, less aromatic molecules: regions 1–9; moderately aromatic molecules: regions 10–18; more aromatic molecules: regions 19–27); lipophilic compounds are situated in the positive direction of PC3 while predominantly polar compounds are located in the negative PC3 direction (that is, polar molecules: regions 1, 2, 3, 10, 11, 12, 19, 20 and 21; mediate-polar molecules: regions 4, 5, 6, 13, 14, 15, 22, 23 and 24; non-polar molecules: regions 7, 8, 9, 16, 17, 18, 25, 26 and 27).

**Figure 3:**
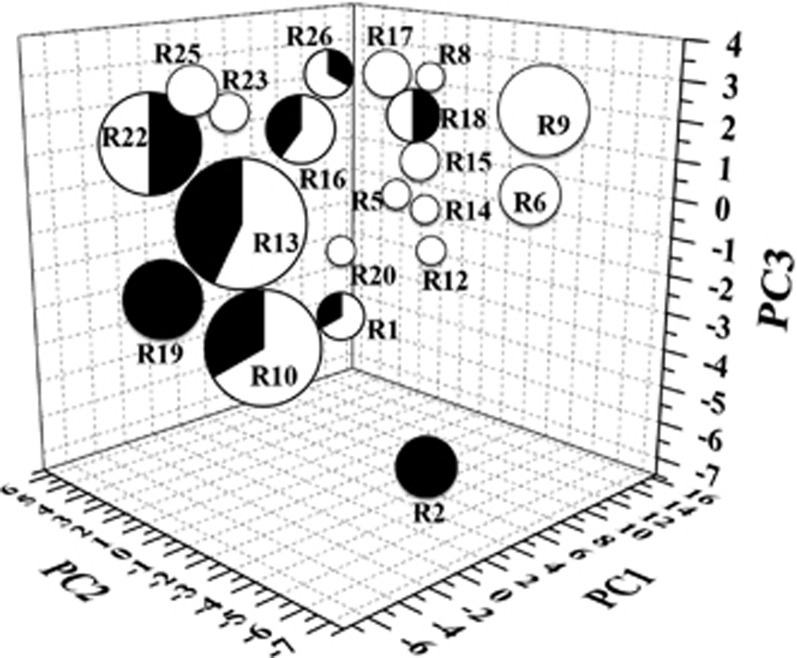
Score plot of mycobacteria-active natural products from marine microbes and TB drugs and candidates in regions. Total numbers of compounds in each region is indicated by sizes of pie charts, percentages of *M*. tuberculosis NPs from marine microbes and TB drugs in each region are showed in white and black, respectively. PC1 (molecular size) versus PC2 (molecular aromaticity) versus PC3 (molecular lipophilicity). TB, tuberculosis.

All of the marine mycobacteria-active microbial natural products were compared with the corresponding TB drugs in the same region (Figure 3). Region 13, representative of low molecular weight, moderate polarity and aromaticity, holds the most active natural products (12) and TB drugs (9) that belong to two different classes distinguished by the mechanisms of DNA inhibition and protein synthesis inhibition.

**Figure 4:**
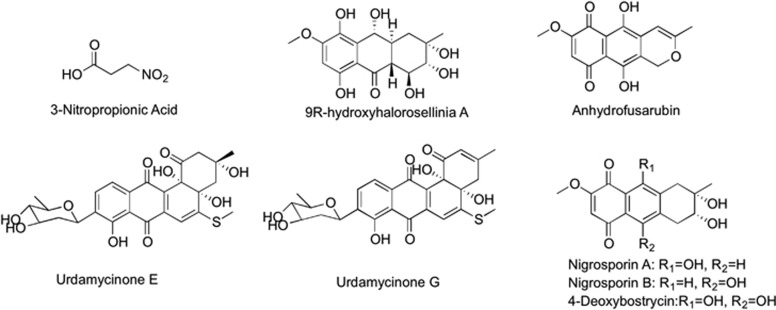
Structures of eight mycobacteria-active natural products in region 10.

The 27 regions can be classified into three classes according to the proportion of TB drugs and mycobacteria-active natural products in each region. Firstly, the injectable and nature-derived TB drugs comprised of streptomycin, kanamycin, amikacin, capreomycin and viomycin clustered together in region 2 and no representative mycobacteria-active natural products occupied this region of physico-chemical space. It is important to note that all of these drugs are protein synthesis inhibitors. Another region that lacked representation of mycobacteria-active natural products from marine microbes is region 19, which contains five cell wall inhibitors (isoniazid, pyrazinamide, thioacetazone, ethionamide and prothionamide) and aminosalicylic acid. The low density of natural products in these regions indicates a promising field for new natural products with similar mode of mechanism with current drugs. Secondly, there were eight regions that had only mycobacteria-active natural products, region 9 in particular, the second biggest region with nine natural products of which four exhibited anti-*M. tuberculosis* MIC <1 μM. These active natural products have unique properties in chemical space, as well as excellent biological activities. Finally, seven regions contained TB drugs, as well as mycobacteria-active natural products, and according to the theory that compounds with similar activity profile and chemical properties often show a similar mode of action, it is possible to predict the putative mode of action of these natural products. For example, region 10 contained TB drugs with small sizes and cell wall inhibition activities, as well as eight marine microbe-derived mycobacteria-active natural products (Figure 4). These natural products may be potential mycobacteria cell wall inhibitors. Similarly, natural products in region 22 and 26 could be predicted as promising cell wall inhibitors and ATP synthesis inhibitors, respectively. Moreover, there were twelve mycobacteria-active natural products and nine anti-TB drugs in region 13, including six DNA inhibitors and three protein inhibitors. Based on the same hypothesis, the mode of mechanisms of these compounds would be predicted as inhibitors of DNA or proteins of mycobacteria. This hypothesis was strongly supported by the fact that nine compounds in region 13 with known mode of mechanisms are all protein inhibitors, consistent with our prediction.

## Natural product near neighbors of approved drugs

Calculation of Euclidean distances based on ChemGPS scores has been found to be a useful tool to identify natural products leads for drug discovery.^15^ The EDs were calculated between points *P*=(*p*_1_, *p*_2_,..., *p*_*n*_) and *Q*= (*q*_1_, *q*_2_,..., *q*_n_) in the Euclidean n-dimensional space, as defined by the following expression:


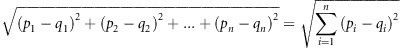


**Figure 5:**
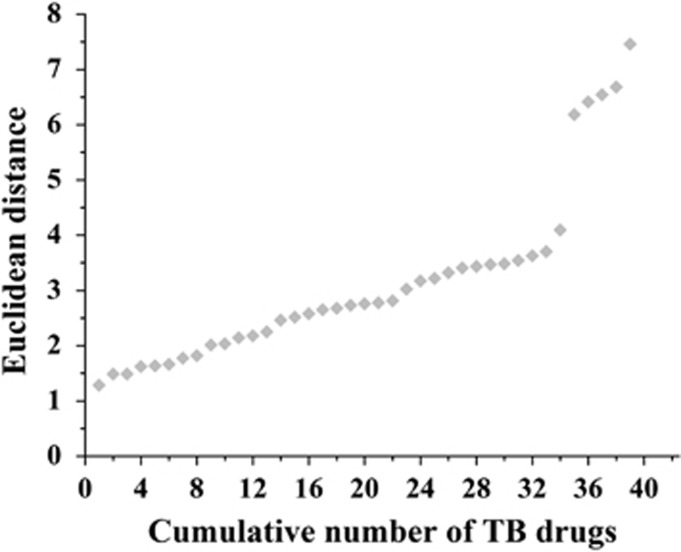
Distribution of ED to the nearest natural product neighbor for the TB drugs and candidates. The cumulative number of drugs is plotted against the ED to the closest natural product neighbor. TB, tuberculosis.

Thereby all mycobacteria-active natural products were assigned with 39 EDs by the use of all eight coordinates calculated by ChemGPS, one ED to each drug. In Figure 5, the 39 drugs are plotted against the ED to their closest natural product neighbor. Interestingly, all drugs have a natural product neighbor closer than ED=8, and around 85% of the drugs have a natural product neighbor closer than ED=4. This forms a strong argument that natural products from marine microbes have the potential to serve as an important source of TB drugs.

**Figure 6:**
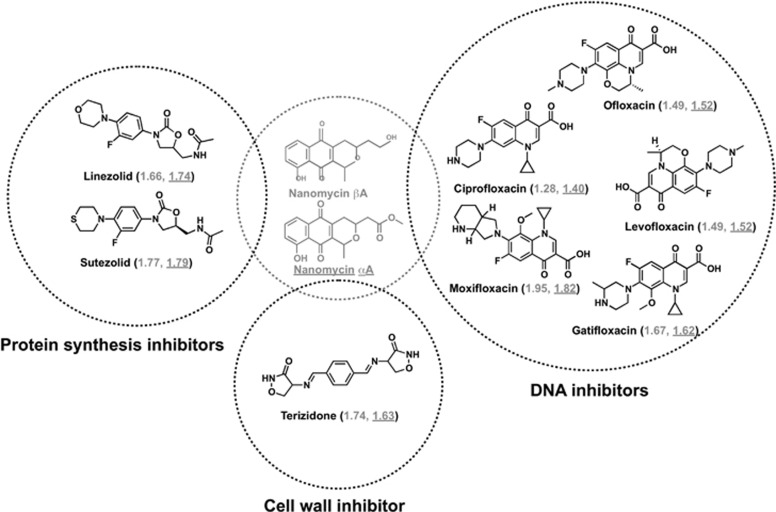
Structures of marine microbe-derived mycobacteria-active natural products nanaomycin αA and nanaomycin βA and anti-TB drugs with EDs shorter than 2. Anti-TB drugs with protein synthesis inhibitor activity are in orange, DNA inhibitors and cell wall inhibitors are in black. EDs between drugs and nanaomycin αA and nanaomycin βA are given in gray without and with underline, respectively. TB, tuberculosis.

Some natural products showed short EDs with more than one drug neighbor (Figure 6). Nanomycin *β*A and *α*A, isolated from a marine-derived *Streptomycetes* sp., have been reported to inhibit mycoplasma, fungi and Gram-positive bacteria, showing inhibitory activity against *M. tuberculosis* H37Rv with an MIC value of 8.0 μg ml^−1^. Nanomycins showed a close relationship with nine anti-TB drugs, including one cell wall inhibitor, five DNA inhibitors and two protein synthesis inhibitors, with ED value <2. We suggest that the natural products with short EDs to known drugs should be paid particular attention in developing new anti-TB drugs candidates because of their high similarity with known drugs in physico-chemical space.

**Figure 7:**
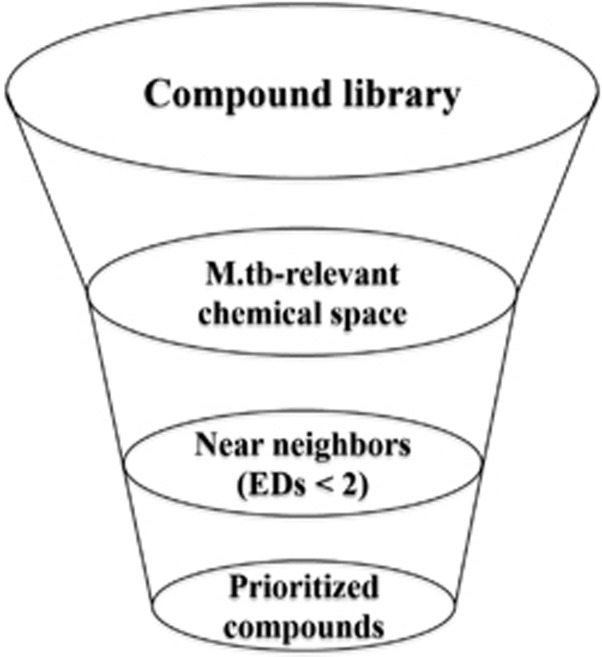
Main factors to design diverse combinatorial libraries or to select diverse compounds to conduct an anti-*M. tuberculosis* screening collection.

The above information can be used to develop a strategy to select compounds when conducting anti-*M. tuberculosis* screening (Figure 7). First, mapping of candidate compounds into the *M. tuberculosis*-relevant chemical space defined by TB drugs and *M. tuberculosis*-active natural products allows for a quick and efficient filter for potential *M. tuberculosis*-activity. The location of compounds in this model can also be used to predict potential mode of action (MoA). Second, compounds with EDs<2 to known TB drugs provides a second filter. Screening of the prioritized compounds in chemical space may directly lead to the identification of biologically relevant structures for TB drug discovery.

## Applying the model

**Figure 8:**
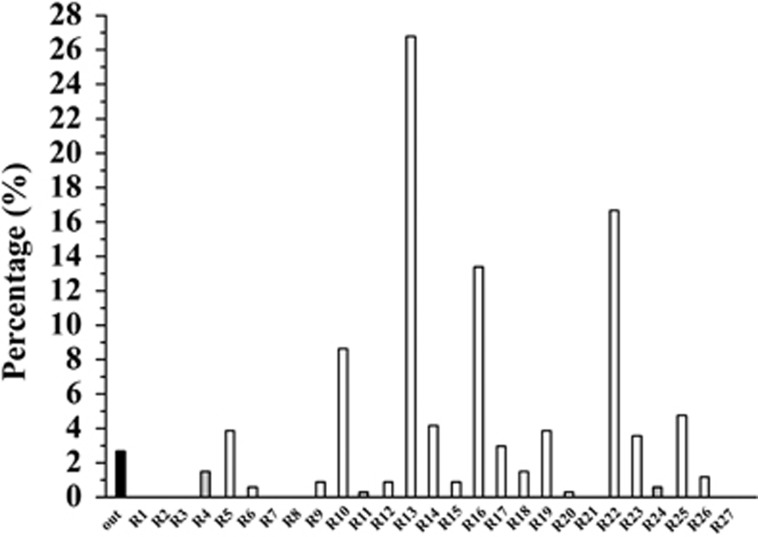
Histogram of the percentage of untested NPs from marine microbes in each regions. Compounds not falling into the 27 regions were plotted in black, compounds in regions 4, 11 and 24 without represented TB drugs and *M. tuberculosis*-active NPs were plotted in gray. TB, tuberculosis.

Data set 3 (Table 1) was analyzed using the model. 327 of 336 compounds were found to fall into the 27-region chemical space (Figure 8). Similar with *M. tuberculosis*-active natural products, region 13 contained the most untested compounds (90). Surprisingly, three regions 4, 11 and 24, which have no represented TB drugs and *M. tuberculosis* active compounds, occupied with 5, 1 and 2 untested compounds, respectively. These compounds may show unique structure properties.

**Figure 9:**
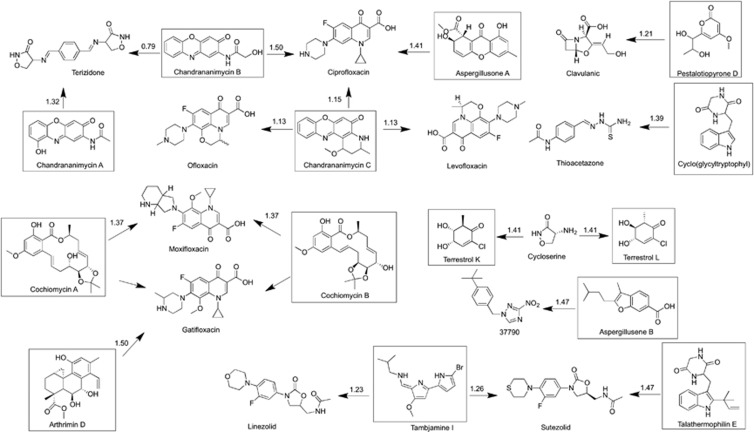
Structures of top 20 shortest EDs of untested natural products/drugs pairs. Untested natural products from marine microbes are in red and TB drugs and candidates are in blue and EDs are given in black numbers. TB, tuberculosis.

Filtering these compounds using EDs calculation to identify near neighbors with known TB dugs produced 82 prioritized structures with ED<2 to at least one drug. Structures of the top 20 nearest EDs of untested natural products/drugs are given in Figure 9. Untested compounds showing short EDs with more than one drug neighbor may be worth screening.

## Conclusion

TB threatens people's lives around the world and the appearance of drug resistance has increased the need to identify novel anti-TB drugs. The complex etiology of the disease involving oral bioavailability, lung-alveolar macrophages, granulomas and mycobacterial cell permeability, requires complex modelling. We used the chemical space navigation tool ChemGPS-NP to compare 60 mycobacteria-active natural products and 39 TB drugs and drug candidates with respect to physico-chemical properties and their occupation of chemical space. In physico-chemical space, both sets largely overlapped and defined a region of Chem-GPS-NP space

Further analysis by ChemGPS-NP defined 27 regions within TB active space. Secondly, EDs to known TB drugs may be a better predictive tool. We concluded that if a compound has a ED of <2 to any of the current TB drugs, then the compound has a much higher chance of itself being active.

## Figures and Tables

**Table 1:** Information of three data sets used in this review

*Data set*	*Data set name*	*Anti-M. tuberculosis activity*	*Number of compounds*	*Compound types*	*Compound sources*
1	TB drugs and candidates	Active	39	Natural products and synthetic	Various
2	Marine microbial NPs	Active	60	Natural products	Marine microbes
3	Marine microbial NPs	Unknown	336	Natural products	Marine microbes

Abbreviations: NPs, natural products; TB, tuberculosis.

**Table 2:** The scores specification of each region

*Regions*	*PC1*	*PC2*	*PC3*
Total score range	−4.24~14.14	−5.72~4.89	−2.84~3.54
Region 1	−4.24~2	−5.72~−2	−2.84~−1
Region 2	2~8	−5.72~−2	−2.84~−1
Region 3	8~14.14	−5.72~−2	−2.84~−1
Region 4	−4.24~2	−5.72~−2	−1~1
Region 5	2~8	−5.72~−2	−1~1
Region 6	8~14.14	−5.72~−2	−1~1
Region 7	−4.24~2	−5.72~−2	1~3.54
Region 8	2~8	−5.72~−2	1~3.54
Region 9	8~14.14	−5.72~−2	1~3.54
Region 10	−4.24~2	−2~1	−2.84~−1
Region 11	2~8	−2~1	−2.84~−1
Region 12	8~14.14	−2~1	−2.84~−1
Region 13	−4.24~2	−2~1	−1~1
Region 14	2~8	−2~1	−1~1
Region 15	8~14.14	−2~1	−1~1
Region 16	−4.24~2	−2~1	1~3.54
Region 17	2~8	−2~1	1~3.54
Region 18	8~14.14	−2~1	1~3.54
Region 19	−4.24~2	1~4.89	−2.84~−1
Region 20	2~8	1~4.89	−2.84~−1
Region 21	8~14.14	1~4.89	−2.84~−1
Region 22	−4.24~2	1~4.89	−1~1
Region 23	2~8	1~4.89	−1~1
Region 24	8~14.14	1~4.89	−1~1
Region 25	−4.24~2	1~4.89	1~3.54
Region 26	2~8	1~4.89	1~3.54
Region 27	8~14.14	1~4.89	1~3.54
